# Progress in Psychiatry

**Published:** 1900-06-16

**Authors:** 


### PROGRESS IN PSYCHIATRY.

(*Continued from page* 173.)

**General Considerations Regarding the Degenerates.**—The civilised man has beneath the thin veneer which a few centuries of civilisation and education have imposed upon him the animal and savage nature inherited from his ancestors for countless generations past. Carlyle well expressed it when he wrote that beneath the thin veneer "the savage nature of man burns perpetually as with an eternal fire," and Tennyson, who understood evolution better than many of his contemporaries, wrote of the "moods of tiger and of ape" lurking within man. Hence it is that society has found it necessary to enact laws against violence, murder and manslaughter, robbery and theft, rape and incendiarism, and the numerous offences and aggressions of which men and women may be the victims at the hands of their fellows. The obligation and duty of the community is to prevent everything that shall make individuals dangerous to themselves or to others. The various classes of lunatics and criminals bring themselves within the category of the dangerous, and therefore require sequestration and special care and supervision. It will be evident, however, that this is a mode of dealing with the question which is scarcely satisfactory or sufficient. The accumulation of these hosts of degenerates in our workhouses, prisons, and asylums goes on year by year, and for many years the question has been asked, "Can anything be done to prevent this increase?" Schemes which a few years ago appeared visionary are now assuming a more realisable and practicable aspect. The sequestration of the confirmed inebriate who has been convicted before a proper judicial authority is now in process of becoming an accomplished fact. The establishment of reformatories for youthful offenders has proved that relatively useful and productive lives may be lived by boys and girls with moral and intellectual defects of moderate grade. The development of farming and industrial colonies for chronic epileptics at Chalfont St. Peter, in England, and at the Craig Colony in New York has justified the hopes of those who sought to render the epileptic capable in some measure of a social and useful life within the limits and under the restrictions of a farming colony. Finally, the recent legal provisions facilitating the certification and treatment of "incipient and unconfirmed" cases of insanity constitute a step towards the possible mitigation of the ravages of mental disease, which, treated thus early, might yield better to treatment, so that in favourable cases patients might be stopped from drifting into the class of the chronic incurable and hopelessly insane. The growth of knowledge and the teachings of experience have led us to important developments as regards the classification and housing of chronic lunatics and epileptics on a large scale on a medico-economic basis. More recently the question of special public provision—on the "hospital" system—for the treatment and cure of the curable insanities has been pressing itself upon county councils and other local authorities. There remains, however, the most important practical question of all, viz., the means and methods for the gradual prevention or limitation of the preventable insanities. It will be seen on casting an eye over the various factors already referred to under the heading Ætiology and Morbid Heredity (Vol. XXVII., pages 211-213) that some of the ætiological factors must be capable of being dealt with more or less successfully. (*a*) Conditions of housing, food, and clothing, and the other evils attending unhealthy dwellings, overcrowding, and destitution, are capable of improvement. (*b*) Alcoholism can be limited in its ravages by education of public opinion, the spread of scientific knowledge, and the enactment of legislation for inebriety, &c. (*c*) The incidence of syphilis both on the military and civil populations might similarly be lessened by the compulsory sequestration of prostitutes suffering from the disease, and by the inculcation of the virtues of chastity and temperance in sexual life. The serious and deplorable increase in the number of patients suffering from general paralysis of the insane in Great Britain as shown by asylum records, and the close ætiological connection it has with syphilis, gives rise to the hope that any serious and practical measure to limit the spread of syphilis would be fraught with the blessing of limiting the production of general paralysis of the insane—the most fatal of the insanities. (*d*) The campaign against tuberculosis is already in active operation, and must be fraught with advantage to the race in the immediate future. (*e*) The marriage of epileptics, or of persons who have had one or more attacks of insanity in youth or early adult life, the intermarriage of deaf-mutes, the marriage of persons with profound hysteria, grave chorea, grave neurasthenia, or with tendencies towards suicidal and other dangerous impulses, are all questions of grave and serious import, and requiring the nicest judgment and discrimination—questions on which the physician's advice may be often asked though not so often followed. The education of public opinion on these points is a foremost duty of the mental physician. The free and unlimited increase of the degenerate, the effete, the neuropathic, and the lunatic tends to constitute a serious and growing danger to the safety of society and to the resources and health of a nation. The nation that turns its attention to eliminate the unfit from its potential fathers and mothers of future generations will be bound to get a long start of its rivals in the race for progress, civilisation, and national well-being. Neither social nor economic progress can be promoted where the citizens and ratepayers are over-burdened with the maintenance of an excessive number of lunatics, criminals, and paupers.

